# 

**Published:** 1887-04-16

**Authors:** 


### CONTENTS.

**Easter Eggs** - - - - - - - 33
**Words of Conso'ation.**—XXIX. Death - - 34
**Poetry.**—Bury Me in the Churchyard at Home - - 34
**Nurse Marion's Romance.** By KATE FURLEY. Chapter IV. The Crisis - - - - - - 35
**Moral and Physical Massage.** By Dr. STRETCH DOWSE - - - - - - - 37
**Questions and Answers.**—Homes for Convalescents with Wounds - - - - - - 38
**The Harley Street Establishment for Invalid Gentlewomen.** By a LADY VISITOR - 39
**Notes and News.** — Miscellaneous Items. — Jubilee "Banner Service."—Entertainment at the Cancer Hospital.—The St. John's Ambulance Association.—Hospital Saturday Fund and the London Cabmen.—Origin of Hospital Sunday.—Hospital Reports.—Donations, etc., to Medical Charities.—Concert in aid of the Miller Hospital.—Lectures.—The Silloth Convalescent Institution. —Hospital Construction, etc., etc. - - - - 40
**Annotations.**—Free Criticism.—The Aberdeen Royal Infirmary.—Amusements in Hospitals - - - 43
**A Word to Hospital Patients.** By ONE OF THEMSELVES - - - - - - 44
**Till the Doctor Comes.** XV. Every-day Ailments. XVI. Trusses and Stockings - - - - - 45
**Early Rising: Cheerful Health.** By Mrs. HYNES 46
**A National Pension Fund for Hospital Officials and Nurses.** The Views of Officials, Sisters, Nurses, and Others - - - - - 47
**The Children's Corner.**—Puzzles, Answers, etc. - 48
**Amusements with Profit** - - - - 48
**In- and Out-Patients' Hospital Diary** - - 49

